# Referral Patterns of Pediatric Patients to Otolaryngology by Primary Care Physicians at a Single Secondary Hospital in Saudi Arabia: A Retrospective Cross-Sectional Study

**DOI:** 10.7759/cureus.83990

**Published:** 2025-05-12

**Authors:** Othman M Alothman, Naif M Alshahrani

**Affiliations:** 1 Family Medicine, King Fahad Medical City, Riyadh, SAU

**Keywords:** diagnostic tools, nasal complaints, otolaryngology referrals, pediatric patients, primary care physicians, referral appropriateness, retrospective study, saudi arabia, secondary hospital, tympanometry

## Abstract

Background

Otolaryngologic conditions are among the most common reasons for pediatric visits to primary health care units. However, many referrals to otolaryngology specialists may be unnecessary, leading to potential overutilization. This study investigates referral patterns, diagnostic practices, and the appropriateness of referrals for pediatric patients at a secondary hospital in Riyadh, Saudi Arabia.

Objective

The objective of this study is to assess the demographics, reasons for referral, diagnostic approaches, and appropriateness of otolaryngology referrals for pediatric patients from primary care physicians.

Methods

A retrospective, cross-sectional analysis was conducted on 336 pediatric otolaryngology referrals (ages ≤14) from 2022 to 2024 at Prince Mohammed Bin Abdulaziz Hospital. Variables included patient demographics, referral reasons, diagnostic tools used, and final outcomes. Appropriateness of referrals was assessed, and statistical tests were performed to identify predictors of appropriate referral.

Results

The sample included 65.2% males and 96.7% Saudi nationals. The most common referral reasons were nasal symptoms (27.7%), throat symptoms (16.7%), and airway symptoms (15.2%). The most frequent diagnoses were adenoid hypertrophy with or without tonsillar disorders. About 31.5% of referrals were deemed inappropriate, with nasal complaints accounting for 73% of those. The use of diagnostic tools significantly increased referral appropriateness (p = 0.002); tympanometry, in particular, was associated with higher accuracy. Age was a significant predictor; children aged six to 10 were more likely to receive appropriate referrals (p < 0.05). No significant association was found between comorbidities and referral appropriateness.

Conclusions

A substantial proportion of pediatric otolaryngology referrals were inappropriate, especially those for nasal symptoms, which were often unsupported by diagnostic evaluation. These findings highlight a need for enhanced otolaryngology training in primary care, better access to basic diagnostic tools, and implementation of standardized referral guidelines to optimize specialist utilization and improve patient care.

## Introduction

Otorhinolaryngology, also known as ENT, is a surgical specialty concerned with conditions affecting the senses of hearing, balance, smell, and taste, as well as issues related to breathing, swallowing, and voice [1]. Otorhinolaryngological complaints represent a significant portion of visits to primary care. Approximately 20% of adult general practice consultations are related to ENT complaints [2]. In a university-based primary healthcare center (PHC) in Saudi Arabia, ENT-related problems accounted for 4.7% of pediatric visits, making it the fifth most common reason for consultation [3].

A US-based study analyzing pediatric referral patterns from PHCs found that ENT, orthopedic surgery, and ophthalmology were the most frequently consulted surgical specialties [4]. Despite the frequency of ENT-related concerns, studies have identified a knowledge gap among primary care physicians regarding ENT presentations. A study conducted in Riyadh found that nearly 70% of surveyed physicians have poor-to-average knowledge of tonsillectomy indications, highlighting the need to strengthen otolaryngology education in medical schools and residency programs [5]. Similarly, research from the University of Washington in Seattle revealed a low level of ENT knowledge among primary care providers attending an otolaryngology update course [6].

Improving the quality of referrals can lead to significant cost savings and improved patient care. A London clinic reduced inappropriate ENT referrals and achieved a cost saving of £32,490 over a three-month period [7]. Moreover, timely and appropriate referrals have been associated with lower healthcare expenditures in managing conditions such as voice disorders [8]. In contrast, delayed, incomplete, or misdirected referrals can result in unnecessary diagnostic testing, delays in treatment, and both physical and financial harm to patients [9].

In Saudi Arabia, limited research exists on medical referral patterns [10]. Referrals are often time-consuming and costly, making it crucial to understand their distribution by region. Doing so can improve access to timely and appropriate care, ultimately enhancing patient outcomes.

Given this context, the present study aimed to (1) analyze the demographic and clinical characteristics of pediatric ENT referrals; (2) assess the appropriateness of referrals, including the role of diagnostic tools; and (3) identify predictors of inappropriate referrals to optimize specialist utilization.

## Materials and methods

This retrospective cross-sectional study was conducted at Prince Mohammed Bin Abdulaziz Hospital, a secondary hospital located in Riyadh, Saudi Arabia. The study covered pediatric referrals to the otolaryngology (ENT) department from January 2022 to December 2024. The hospital receives referrals from PHCs affiliated with the Riyadh Second Health Cluster and serves both Saudi and non-Saudi nationals.

The study population included all pediatric patients aged 14 years or younger who were referred to the ENT department during the study period. Inclusion criteria were (1) age ≤14 years; (2) either sex; (3) any nationality; and (4) availability of complete referral documentation. Exclusion criteria included incomplete or missing documentation, missed appointments, or loss to follow-up. Sampling was non-probability consecutive, including all eligible referrals during the study period. Of 459 referrals, 123 (26.8%) were excluded due to incomplete documentation or referrals originating from non-primary care departments. These objective exclusion criteria helped minimize potential selection bias and ensured data quality.

Data were extracted from the hospital’s electronic medical record system (CERNER) using a standardized data collection sheet by three trained data collectors. To ensure consistency and reliability in data extraction, inter-rater reliability was assessed by having each data collector independently extract data from a random subset of 10% of the records. Discrepancies between the data collectors were reviewed and resolved through discussion. Variables included patient demographics (age, sex, and nationality), reason for referral, clinical symptoms, diagnostic tools used (e.g., tympanometry and imaging), and final specialist assessment.

Referral appropriateness was determined using predefined clinical criteria informed by established local primary care guidelines. A referral was classified as appropriate if it necessitated specialist diagnostic procedures (e.g., endoscopic examination and tympanometry) or required advanced therapeutic interventions such as surgical management or complex, multidisciplinary care. In contrast, referrals were deemed inappropriate if the condition could be effectively managed within the scope of primary care without specialist input; examples include uncomplicated rhinitis, cerumen impaction, or recurrent pharyngitis without alarming features.

Data were entered into Microsoft Excel and analyzed using IBM SPSS Statistics for Windows, Version 26.0 (Released 2019; IBM Corp., Armonk, NY, USA). Descriptive statistics were used to summarize the data. Associations between variables and referral appropriateness were tested. A p-value of <0.05 was considered statistically significant.

All patient information was anonymized to maintain confidentiality, and informed consent was waived due to the retrospective nature of the study. Ethical approval was obtained from the Institutional Review Board of King Fahad Medical City, Riyadh, Saudi Arabia (approval number 24-565).

## Results

**Figure 1:**
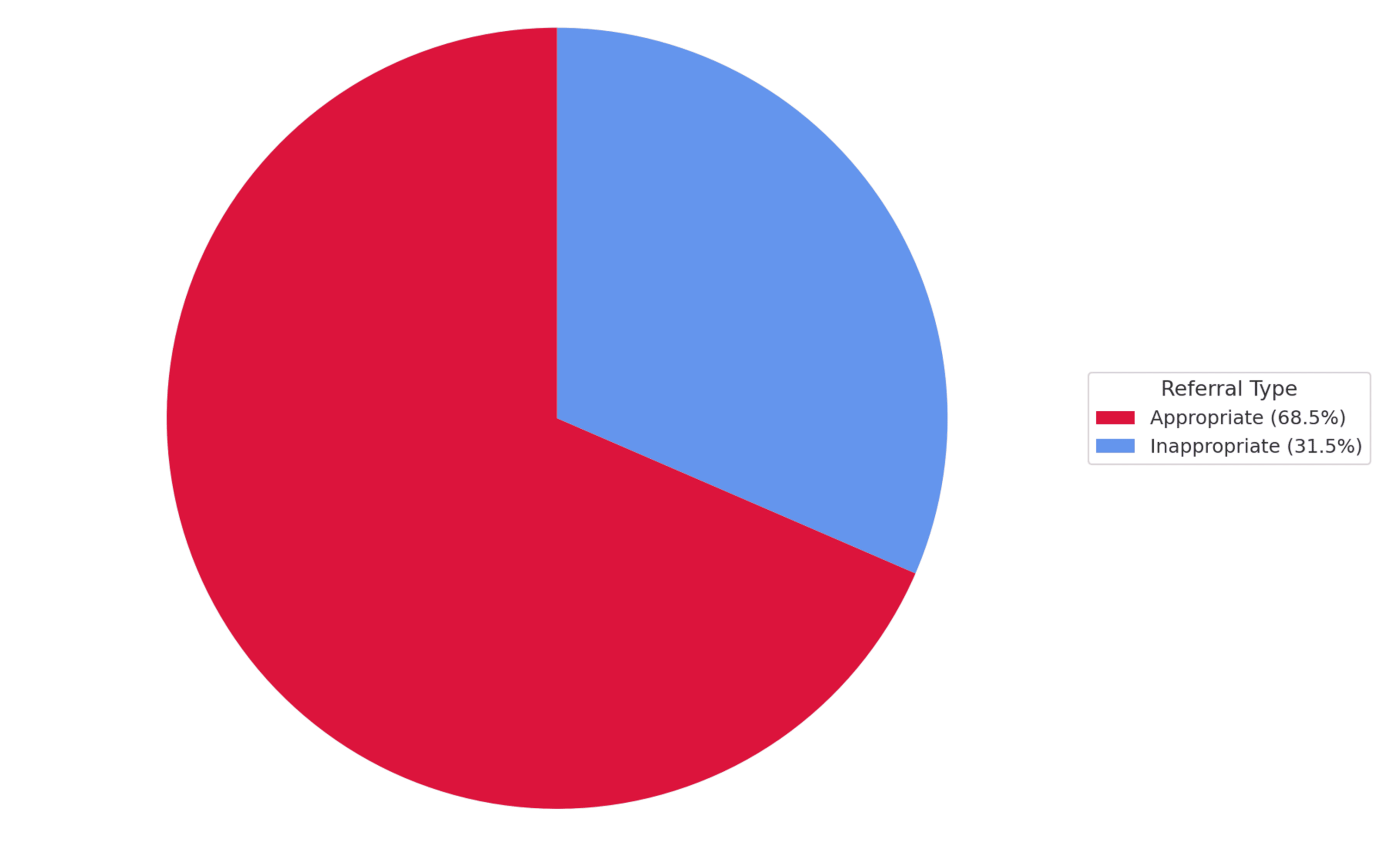
Distribution of appropriate vs. inappropriate pediatric ENT referrals (N = 336)

A total of 336 pediatric ENT referrals were analyzed. The sample consisted predominantly of male patients (219; 65.2%) and Saudi nationals (325; 96.7%), with a mean age of 9.6 years (SD = 2.65). Nasal symptoms (93; 27.7%), throat symptoms (56; 16.7%), and airway symptoms (51; 15.2%) were the most frequent referral reasons. The top diagnoses included adenoid hypertrophy with tonsillar disorders (74; 22.0%) and isolated tonsillar disorders (55; 16.4%). Common interventions were nasal sprays (100; 29.8%) and combined adenoidectomy and tonsillectomy (68; 20.2%). Most referrals were appropriate (230; 68.5%), while 106 (31.5%) were inappropriate (Figure 1). Further demographic and clinical characteristics are detailed in Table 1.

**Table 1:** Descriptive statistics for demographics ^*^ Tonsillar disorders included conditions such as tonsillitis, tonsillar hypertrophy, peritonsillar abscess, and other related disorders of the tonsils.

Variables	Category	N (%)
Sex	Male	219 (65.2%)
	Female	117 (34.8%)
Nationality	Saudi	325 (96.7%)
	Non-Saudi	11 (3.3%)
Referral reason	Nasal symptoms	93 (27.7%)
	Throat symptoms	56 (16.7%)
	Airway symptoms	51 (15.2%)
	Combined airway and throat symptoms	50 (14.9%)
	Ear symptoms	48 (14.3%)
	Other (speech, structural, etc.)	38 (11.3%)
Top diagnoses	Combined adenoid hypertrophy and tonsillar disorders	74 (22.0%)
	Tonsillar disorders^*^	55 (16.4%)
	Adenoid hypertrophy	39 (11.6%)
	Inferior turbinate hypertrophy	33 (9.8%)
	Allergic rhinitis	23 (6.8%)
Intervention	Nasal sprays	100 (29.8%)
	Combined adenoidectomy and tonsillectomy	68 (20.2%)
	Tonsillectomy	44 (13.1%)
	None	40 (11.9%)
	Other (each <5%)	84 (25.0%)
Diagnostic tools used	None	186 (55.4%)
	Tympanometry	80 (23.8%)
	Nasal endoscopy	26 (7.7%)
	X-ray	20 (6.0%)
	Others (each <3%)	24 (7.1%)
Referral appropriateness	Appropriate	230 (68.5%)
	Inappropriate	106 (31.5%)

**Figure 2:**
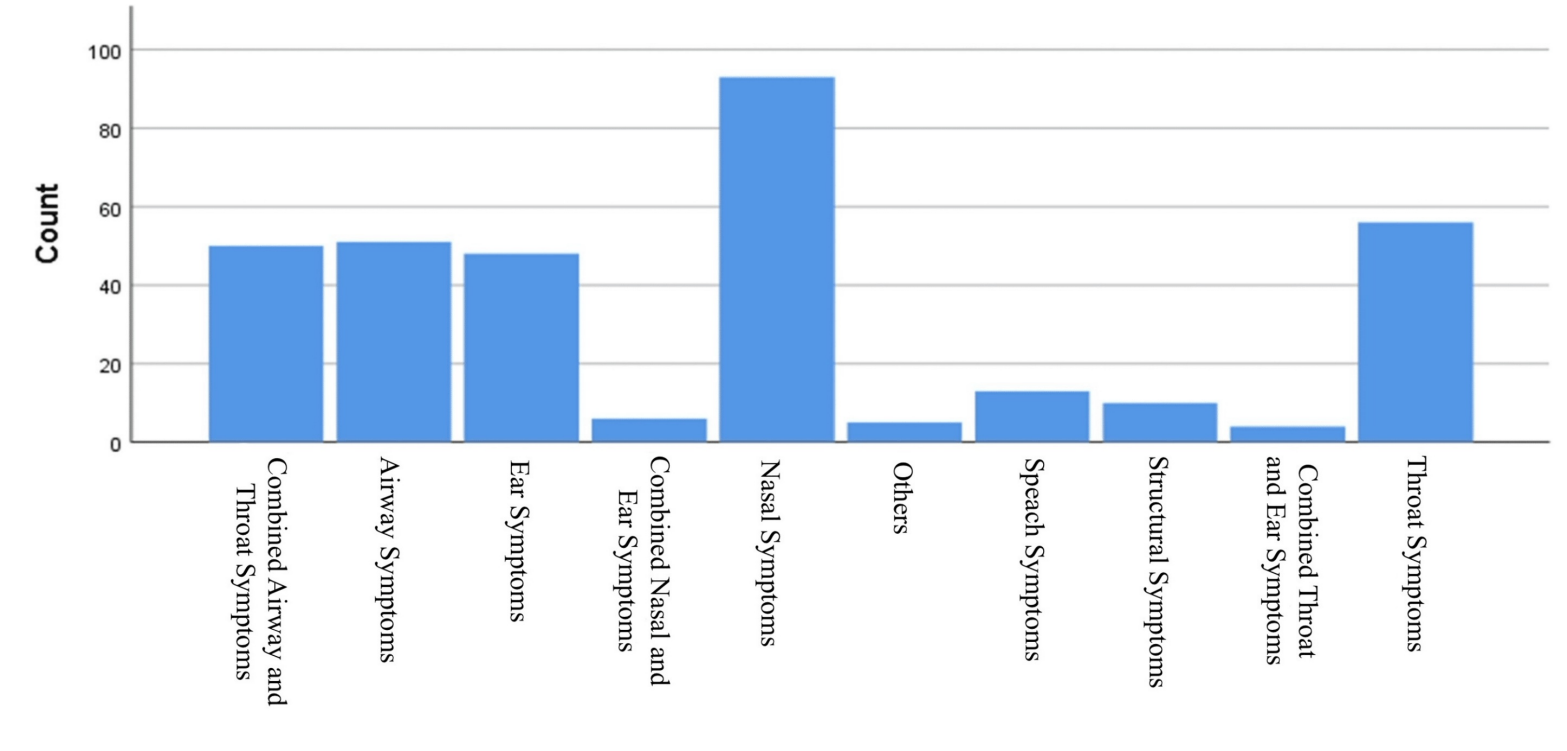
Frequency of referral reasons in pediatric ENT cases (N = 336)

As shown in Figure 2, nasal symptoms were the most common referral reason (93; 27.7%), followed by throat symptoms (56; 16.7%) and airway symptoms (51; 15.2%). Less frequent reasons included ear-related problems (48; 14.3%), speech issues (13; 3.9%), and structural anomalies (12; 3.6%).

A significant association was observed between referral reason and appropriateness (p < 0.001). Nasal symptoms had the highest rate of inappropriate referrals (68 (73.1%) out of 93). In contrast, referrals for throat symptoms were appropriate in 52 (92.9%) of 56 cases, speech symptoms in 13 (100%) of 13, and combined airway and throat symptoms in 48 (96.0%) of 50 cases (Table 2).

**Table 2:** Association between referral reason and appropriateness

Referral reason	Inappropriate, n (%)	Appropriate, n (%)
Airway symptoms	18 (35.3%)	33 (64.7%)
Combined airway and throat symptoms	2 (4.0%)	48 (96.0%)
Ear symptoms	7 (14.6%)	41 (85.4%)
Nasal symptoms	68 (73.1%)	25 (26.9%)
Combined nasal and ear symptoms	2 (33.3%)	4 (66.7%)
Other symptoms	0 (0%)	5 (100%)
Speech symptoms	0 (0%)	13 (100%)
Structural symptoms	5 (50.0%)	5 (50.0%)
Throat symptoms	4 (7.1%)	52 (92.9%)
Combined throat and ear symptoms	0 (0%)	4 (100%)

Referrals utilizing diagnostic tools demonstrated higher appropriateness (p = 0.002). Tympanometry was associated with 70 (87.5%) appropriate referrals out of 80, while referrals without diagnostic tools had lower appropriateness, with 113 (60.8%) out of 186. All referrals supported by CT, culture, or ultrasound were appropriate (Table 3).

**Table 3:** Impact of diagnostic tools on referral appropriateness

Diagnostic tool	Inappropriate, n (%)	Appropriate, n (%)
CT	0 (0%)	5 (100%)
Culture	0 (0%)	2 (100%)
None	73 (39.2%)	113 (60.8%)
Nasal endoscopy	9 (34.6%)	17 (65.4%)
Tympanometry	10 (12.5%)	70 (87.5%)
Ultrasound	0 (0%)	2 (100%)
X-ray	10 (50.0%)	10 (50.0%)

## Discussion

This study provides important insight into pediatric otolaryngology referral patterns at a secondary hospital in Saudi Arabia, revealing that nearly one-third (31.5%) of referrals were inappropriate. Nasal symptoms, the most common reason for referral, were also the most frequently deemed unnecessary, with nearly three-quarters found to be inappropriate. These findings align with previous literature indicating that primary care providers may lack the specialized knowledge needed to effectively evaluate ENT conditions [5]. In resource-limited settings, referral error rates may be even higher due to diagnostic constraints. Lukama et al. observed that insufficient diagnostic resources in a low-resource hospital contributed to a misdiagnosis rate exceeding 67%, with over half of ENT referrals deemed inappropriate as a result [11]. This rate is consistent with findings in other settings; for instance, a UK audit reported roughly one-third of general practice ENT referrals to be avoidable, underscoring the need for clearer referral criteria [12]. These comparisons highlight how available resources and referral guidelines can significantly impact referral appropriateness.

Our findings highlight the overrepresentation of nasal complaints in inappropriate referrals, likely due to overlapping symptomatology with more serious conditions and limited access to confirmatory diagnostics in PHC. Primary care providers may default to caution without tools such as nasal endoscopy or imaging. Training and access to such tools could empower clinicians to better distinguish benign conditions from those requiring ENT input. Notably, referrals supported by diagnostic investigations, especially tympanometry, were significantly more appropriate. However, this likely reflects an association rather than a causal relationship, as tympanometry may serve as a proxy for more thorough clinical assessments by referring providers. This supports integrating simple, accessible diagnostics into routine PHC practice to optimize referral accuracy.

Patients who underwent tympanometry were nearly six times more likely to be appropriately referred, underscoring the diagnostic value of such tools in primary settings. Nevertheless, 55.4% of referrals were made without using any diagnostic tool, suggesting underutilization of available resources. This represents a key area for intervention through continuing medical education and equipment availability. Age was also a significant factor: children aged six to 10 years were more often appropriately referred, possibly reflecting clearer symptom patterns or greater provider confidence in this age range. Interestingly, comorbidities did not influence referral appropriateness, indicating potential gaps in risk stratification during referral decision-making.

These findings are consistent with international research emphasizing the clinical and financial implications of inappropriate referrals [7,8]. Unwarranted referrals can overburden specialty services and contribute to unnecessary patient anxiety, lost time, and financial costs. Conversely, missed or delayed referrals may result in adverse health outcomes due to postponed diagnoses and interventions [13].

The need for well-defined referral guidelines is clear. Establishing and adhering to specific criteria can markedly improve referral quality, as evidenced by prior studies. Mahalingam et al. emphasized that educating referring providers on referral criteria is crucial after finding a high proportion of inappropriate ENT clinic referrals [12]. Furthermore, implementing structured referral protocols has proven effective in practice. Mylvaganam et al. reported that introducing guideline-based referrals in an ENT clinic setting reduced the rate of inappropriate referrals from 7% to just 2% [14]. This dramatic improvement illustrates the potential benefits of standardized referral pathways and compliance with recommended criteria.

Overall, our results call for a multifaceted approach to enhance ENT referral accuracy. Clear referral guidelines, improved primary care training, and greater access to diagnostic tools could collectively reduce unnecessary specialist referrals. Such measures, as supported by the literature [11-14], would not only decrease the burden on pediatric ENT services but also ensure patients receive appropriate care at the right level. Maintaining open communication and feedback between ENT specialists and primary care providers may further refine the referral process, ultimately improving patient outcomes.

Limitations

This study has several limitations. It was conducted at a single institution, limiting generalizability. Referral appropriateness was determined using available clinical data and a specialist judgment, which may involve subjective bias. Additionally, the lack of long-term follow-up prevented outcome analysis for patients not referred or inappropriately referred. Future research should include multicenter studies and examine the effects of guideline-based interventions and provider training on improving referral appropriateness in pediatric otolaryngology.

## Conclusions

This study reveals a high rate of inappropriate referrals to pediatric ENT services, particularly for nasal complaints, and highlights a substantial underuse of diagnostic tools in primary care. Key factors associated with appropriate referrals included the use of tympanometry and patient age, while comorbidities showed no significant impact. These findings suggest that limited diagnostic support and clinical uncertainty may drive unnecessary specialist referrals.

To address these gaps, efforts should focus on reinforcing ENT-related education in PHC training programs, promoting the use of basic diagnostic tools, and implementing clear, evidence-based referral protocols. Such measures may reduce unnecessary specialist visits, improve referral accuracy, and optimize healthcare resource utilization. Furthermore, the development of targeted policies or clinical guidelines that emphasize the importance of appropriate diagnostic support could further enhance referral practices. Future research should assess the effectiveness of these interventions across broader, multi-center populations.
